# Cognitive Impairment and Celiac Disease: Is Transcranial Magnetic Stimulation a *Trait d’Union* between Gut and Brain?

**DOI:** 10.3390/ijms19082243

**Published:** 2018-07-31

**Authors:** Giuseppe Lanza, Rita Bella, Mariagiovanna Cantone, Giovanni Pennisi, Raffaele Ferri, Manuela Pennisi

**Affiliations:** 1 Oasi Research Institute-IRCCS, Via Conte Ruggero, 73-94018 Troina, Italy; rferri@oasi.en.it; 2 Department of Medical and Surgical Sciences and Advanced Technologies, Section of Neurosciences, University of Catania, Via S. Sofia, 78-95123 Catania, Italy; rbella@unict.it; 3 IRCCS Centro Neurolesi Bonino Pulejo, Via Provinciale Palermo, Contrada Casazza, 98124 Messina, Italy; mariagiovanna.cantone@irccsme.it; 4 Department of Surgery and Medical-Surgery Specialties, University of Catania, Via S. Sofia, 78-95123 Catania, Italy; pennigi@unict.it; 5 Spinal Unit, Emergency Hospital Cannizzaro, Via Messina, 829-95126 Catania, Italy; manuelapennisi78@gmail.com

**Keywords:** celiac disease, cortical excitability, motor evoked potentials, neuroplasticity, gut-brain axis

## Abstract

Celiac disease is a systemic disorder with multifactorial pathogenesis and multifaceted symptomatology. In response to gluten exposure, a significant part of the general population produces antibodies that have been hypothesized to be deleterious to the brain. Among the well-known neurological manifestations, adult celiac patients often complain cognitive symptoms, ranging from the so-called “brain fog” till an overt dementia. Transcranial magnetic stimulation (TMS) is a non-invasive brain stimulation technique that can contribute to the assessment and monitoring of celiac patients, even in those without a clear neurological involvement. The studies here reviewed seem to converge on an impaired central motor conductivity and a “hyperexcitable celiac brain” to TMS, which partially reverts back after a long-term gluten restriction. Notably, a clear hyperexcitability is a stably reported feature of both degenerative and vascular dementia. Therefore, given its potential neuroprotective effect, the gluten-free diet should be introduced as early as possible, although the overall response of neurological symptoms (and cognition in particular) is still controversial. Identifying new and possibly modifiable risk factors may be of crucial importance for patients, clinicians, and researchers.

## 1. Introduction

Celiac disease (CD) is an autoimmune disease triggered by the gluten exposure among genetically predisposed people, leading to the small intestine damage and subsequent malabsorption [1,2]. It is estimated that about 0.3–1.5% of Americans and Europeans suffer from CD [3], with most patients being carriers of either DQ2 or DQ8 of histocompatibility complex class II human leukocyte antigen (HLA) haplotype [1], although other non-HLA regions have been shown to be also involved [4]. The main auto-antigen in CD is the tissue transglutaminase (tTG), whereas gut histopathology typically reveals small bowel mucosal villi atrophy [5]. Diagnosis is centred in clinical suspicion, which is later confirmed through duodenal biopsy, laboratory testing, and, in some instances, genetic tests. Currently, the only known treatment is a lifetime gluten-free diet (GFD), which relieves patients from a number of clinical manifestations, decreases the level of serological markers and the chances of malignant and non-malignant related complications [6,7]. While gastro-intestinal symptoms are revealed during the onset of the disease or its early stages in both adult and paediatric patients, they do not appear often as in the past [8]. A number of adult patients manifest various extra-intestinal involvement, even in those without typical gastro-intestinal symptoms [9,10]. From these observations, CD should be considered as a complex systemic disorder with multifaceted symptomatology and multifactorial pathogenesis. 

Latest research shows that about 12% of the general population generates anti-gliadin antibodies (AGA) following gluten consumption [11,12,13]. AGA are thought to be pathological to brain and neurological complications have frequently been reported among celiac patients. These manifestations are either present during the disease onset or may follow after CD appearance [14]. Moreover, there may be silent neurological complications during disease progression and the contrary case is also true. This means that there may be pure peripheral and/or central nervous system (CNS) complications and gut histopathological changes in celiac patients without intestinal symptoms [9]. It is therefore important to confirm if these antibodies might result in a subclinical injury to the brain in such significant proportion of apparently healthy people. This gut-associated damage to the brain would possibly be “concealed” in the medium/short term, meaning that the individuals afflicted will fail to be diagnosed and receive treatment with GFD, leading to cerebral pathology accumulating progressively [15]. When combined, the scenarios depicted show the significance of a diagnostic tool effective for the early detection of any salient progression or presence of CD and its complications.

## 2. Cognitive Complications of Celiac Disease: A Brief Overview and Proposed Mechanisms

All complications due to gluten intake can result in varying degree of brain damage. For instance, serious pathology is manifested in gluten ataxia, which is depicted by a significant loss of Purkinje cells [12,13]. While poorly understood, CD also results in brain atrophy and changes in white matter [8,9,10]. As a result, among the other well-known neurological manifestations (such as peripheral neuropathy, epilepsy, and ataxia), adult celiac patients often present with mild cognitive symptoms known as “brain fog” [16]. The most common features reported include attention and concentration difficulties, word-retrieval challenges, episodic memory deficiency, disorientation or confusion episodes, and declined mental acuity. In addition, various forms of dementia, including vascular dementia (VaD), Alzheimer’s disease (AD) and frontotemporal dementia, have been shown to be associated with CD [17,18], although a direct link is yet to be known. For these reasons, attention, memory, executive functions, and visuospatial abilities need to be screened among every patient with CD, both during the disease progression and at the time of diagnosis. 

A number of mechanisms have been put forward to account for the deleterious effect of gluten-associated diseases on cognitive abilities, including nutritional deficiencies, regional cerebral hypoperfusion, increased levels of circulating cytokines caused by systemic inflammation, and low levels of brain serotonin [19,20,21]. In general, however, limited research has dealt with this topic extensively. It is already known that AGA tends to react with the brain blood vessels [22], thus contributing to the findings from an extensive epidemiological investigation showing that CD results in an increased risk of dementia [23]. Additionally, CD displays a cross-reactivity with the neuronal synapsin 1 that regulates the release of neurotransmitters, suggesting that the antibody has the potential to disrupt the functions of the brain outside its vasculature [24]. At the same time, studies have associated AGA with increased depression rates among patients with and without the disease [25,26]. Therefore, there is a strong evidence to suggest that AGA positivity results in brain pathology, either by a proinflammatory state induction or by cross-reactivity phenomena. In addition, it is known that anti-tTG6 antibodies are frequently the pathogenic trigger for neurological manifestations in CD [9,27]. To date, however, there is no pathognomonic neuroradiological sign of CD, although cerebellar calcifications may be found in some cases associated with temporal lobe epilepsy [28]. Moreover, it has been reported that white matter lesions in CD tend to resemble those of VaD, which celiac patients have recently been shown to have increased risk of [18]. 

Concerning treatment, cognitive and mood improvement was shown to occur only after a long-term GFD (>5 years) [16,29,30], indicating the rationale of prolonged gluten restriction on extra-intestinal symptoms of CD as well. Consequently, GFD should be administered as early as possible after the disease onset, given its potential neuroprotective effect. Undeniably, indeed, AGA levels tend to decrease when individuals take less gluten [31] and GFD has the potential to stop the development of other gluten-associated pathologies [9,10,32,33]. Nonetheless, the effect of GFD still remains a debatable issue and patients >65 years old present with worse cognitive performance compared to sex-and age-matched controls even after a long-term treatment with GFD [34].

## 3. Transcranial Magnetic Stimulation

### 3.1. Short Description and Rationale

Transcranial magnetic simulation (TMS) is a safe and non-invasive neurophysiological technique specifically able to determine the conductivity along the cortico-spinal tract and the excitability of the primary motor cortex (M1) [35]. Today, however, TMS goes well beyond the simple assessment of the pyramidal tract. Albeit TMS is not disease-specific, it is a useful tool that provides an *in vivo* and a real time estimation of the neurochemistry and pathophysiology underlying several psychiatric and neurological disorders. In both instances, the study of the excitability state of the human brain helps in understanding the function of intracortical neurons, callosal fibres, and cortico-subcortical loops [35,36,37,38,39,40,41,42,43,44,45], as well as in providing useful insights on the plasticity-based interventions [46,47,48], including cognitive rehabilitation [49]. Thus, TMS becomes a tool well suited for monitoring and exploring any motor system impairment during the pre-clinical phase of different neuropsychiatric disorders [50] or systemic diseases that affect the CNS, such as CD [51,52,53,54,55]. Additionally, when used together with other neurophysiological (namely, electroencephalography—EEG) or structural/functional imaging techniques, TMS allows the examination of the connectivity between motor and non-motors regions on the brain [56,57]. Lastly, because the technique could be used to investigate the effects of certain drugs that are both agonists or antagonists for a particular neurotransmitter, TMS is used to test for the specific activity of glutamatergic, cholinergic, and gamma-aminobutyric acid (GABA) ergic central circuits (the so called “pharmaco-TMS”) [58,59]. 

In recent time, the TMS-driven parameters are mostly applied in the exploration of the regulatory process of non-motor cerebral areas, thus helping in disentangling the complex cortical networks the interconnect different cerebral regions with motor areas other than M1. Other influenced regions include the cingulate cortex [60], the ventral and dorsal premotor cortices and supplementary motor area, together with other non-motor areas [47]. In particular, there is supporting evidence that the cingulate cortex and the dorsolateral prefrontal cortex are central to mood regulation and cognition [61]. Equally, repetitive TMS (rTMS) of the primary somatosensory cortex (S1) can regulate the central sensory processing, and changes of S1 excitability may influence the neural mechanisms related to M1 functioning and motor control [62,63].

### 3.2. TMS Measures of Interest

A single TMS pulse applied to M1 triggers a motor evoked potential (MEP) when the contralateral muscles are analysed, hence making it possible to non-invasively assess the cortico-spinal conduction [64]. Specifically, the central motor conduction time, which is explained as a difference in latency between MEPs by M1 stimulation and the latency caused by a motor root stimulation, is considered as an index of integrity of the cortico-spinal pathway [35]. MEPs are produced by the indirect activation of the pyramidal cells, through cortico-cortical connections from the main source of inputs to the cortico-spinal cells represented by layer 2/3 and pyramidal neurons. For that reason, MEP amplitude reflects the balance between excitatory and inhibitory intracortical circuits, together with the excitability of the intracortical projections to the cortico-spinal neurons till to the peripheral nerves and muscles [65]. This is particularly true at higher stimulus intensities that produce a more prolonged activation of the cortical networks, thus resulting in a high frequency repetitive discharge of cortico-spinal cells [65].

The resting motor threshold (rMT) is considered a basic parameter in providing global excitation of a central core of M1 neurons [66]. Resting MT is increased by drugs blocking the voltage-gated sodium channels, where the same drugs may not have an effect on GABA. On the other hand, rMT is reduced by drugs increasing glutamatergic transmission not mediated by the N-methyl-D-aspartate (NMDA) receptors, suggesting that rMT reflects both neuronal membrane excitability and non-NMDA receptor glutamatergic neurotransmission [59]. Moreover, MT increases when a substantial portion of the cortico-spinal tract is damaged (i.e., stroke), while decreases when the cortico-spinal tract is hyperexcitable [35]. 

When the single magnetic pulse is delivered during a voluntary contraction of the contralateral target muscle, MEP is followed by a suppression of the electromyographic activity [66]. The phenomenon is identified as the cortical silent period (CSP), which is a measure of the suppression of the cortico-spinal output at a cortical level, probably due to the activation, after an early spinal phase (first 50–75 ms), of inhibitory cortical interneurons mainly mediated by GABA-B transmission [59]. As known, interindividual differences and the inter-session variability of the CSP duration may be large, highlighting the importance of a standardized method of recording and analysis [67]. 

Both excitability and inhibitory activity in the human cortex can be investigated in a non-invasive way with the paired-pulse TMS paradigm, in which a “conditioning stimulus” (subthreshold) is followed by a “test stimulus” (suprathhreshold) [68,69]. Comparing the interval between the pair of TMS pulses (the interstimulus interval—ISI), several measures used to explain the intracortical interactions have been developed. At an ISI of 1–4 ms, the conditioning stimulus results in reduced MEP amplitude, and is named short-latency intracortical inhibition (SICI); at longer ISI (7–20 ms), the effect is the increased amplitude of the motor responses, called intracortical facilitation (ICF) [68,69]. SICI may be explained as an effect of the excitability of the intracortical GABA-A interneurons. In contrast, ICF is a more complex phenomenon, probably related to the activation of a cortical circuit projecting upon cortico-spinal cells different from those preferentially activated by single pulse TMS, and it is probably composed of cells with less pronounced oscillatory properties. ICF is considered as dependent from circuitries involving glutamatergic excitatory interneurons, although it is also regulated by varying transmission pathways and modulated by some subcortical inputs [58,59].

## 4. Data Source and Selection

A search on PubMed with the keywords “celiac disease” with “motor evoked potentials,” “cortical excitability”, and “transcranial magnetic stimulation,” shows 20 results. From these articles, more were excluded by leaving out EEG or other evoked potentials in CD because such articles did not fit within the scope of this review. Furthermore, studies that were done on animals, studies that are not supported by statistical findings, non-English papers, and articles that are not research studies (i.e., commentaries, letter, editorials, reviews, etc.), were not included in the search. Articles that are listed in the references were reviewed in search for more data. After this process, five peer-reviewed publications were found (Table 1).

## 5. TMS Studies in Celiac Disease 

### 5.1. Pure Central Motor Conductivity Studies

Cortico-spinal signs were first reported after engaging 7 of 28 patients with gluten ataxia (Babinski sign in one and brisk tendon reflexes in six), together with the neuropathologic finding of lymphocytic infiltration to the posterior and cerebellum columns. The authors suggested an immune-mediated neurotoxicity, although MEPs were not performed and the impact of GFD was not tested [70]. 

In 1999, Pellecchia and colleagues first applied TMS in a celiac patient. The researchers described the clinical and electrophysiological improvement of ataxia and peripheral neuropathy associated with subclinical CD after two years of GFD. At baseline, they proved that the reduced MEP amplitude of the rectus femoris muscle improved with a proper diet, although motor responses were still not detected in the anterior tibialis muscle [51]. It was supposed that AGA is a possible pathogen, which may be directly or indirectly neurotoxic. 

One year later, the delayed motor response in the left tibialis anterior muscle and abnormal cortical inhibition were reported in one of the two patients diagnosed with CD and cortical myoclonus [52]. The author speculated that, despite the neurophysiological evidence of cerebral cortical involvement, the resulting hyperexcitability was mainly located in the cerebellum and the effects on the sensory-motor cortex represented a remote influence from cerebellar pathology [52].

### 5.2. TMS in De Novo CD Patients

Systematic TMS studies before and after GFD were recently published, based on the evidence that gluten-mediated immune response in CD was associated with the neuropsychiatric manifestations and that the glutamic acid decarboxylase (GAD) antibodies might react with the GABAergic synapses, thus possibly affecting the inhibitory interneurons activity [71,72,73,74]. The objective of this first study was indeed to determine the functional status of the facilitatory and inhibitory intracortical circuits to single and paired-pulse TMS in 20 de novo CD patients without apparent clinical neurological involvement and 20 age-matched controls [53]. Compared to the healthy subjects, celiac patients revealed a pattern towards hyperfacilitation (increased ICF) and disinhibition (reduced SICI) of M1. Such results suggest a subclinical involvement of both glutamatergic and GABAergic systems in neurologically asymptomatic patients. In particular, the cross-reaction between specific neuronal antigens and AGA, together with altered ions levels related to the tTG6-immunoglobulin deposition in the neuronal cells membranes, might have affected the normal balance between inhibitory and excitatory synaptic excitability [53]. Equally, antibodies synthesized with the CNS and directed against GAD may disrupt the normal functioning of the GABAergic interneurons [53,75]. 

### 5.3. TMS in CD after a Short Gluten-Free Diet

Using the hypothesis that the dietary restriction might restore the balance between intracortical excitatory/inhibitory circuits in CD, the same subjects were examined after a short time of GFD (median 16 months) [54]. Gastrointestinal symptoms improved but an expected rise of the cortical excitability to TMS was observed, as indexed by a decrease of rMT. The authors hypothesized that the hyperexcitability might be caused by an immune-mediated dysregulation of the inhibitory GABAergic interneurons. Additionally, the results were explained by a parallel glutamate-mediated cortical functional reorganization compensating for disease progression despite GFD [54]. Notably, the α-amino-3-hydroxy5-methyl-4-isoxazoleproprionic acid (AMPA) type ionotropic glutamate receptor is vital for excitatory and plasticity synaptic transmission at many levels of the post-synaptic membranes [76], including M1 [77]. Hence, it cannot be excluded that the receptor activation, triggered by gluten-related immune system dysregulation, might lead to an increase of the excitatory post-synaptic potentials underlying the motor cortex hyperexcitability and possibly associated with the phenomena of long-term plasticity. The finding was hypothesized to occur even without a direct role of GFD [54]. 

However, the period of GFD may have not been sufficient to induce an adequate remission. Accordingly, although obtained from a small sample size, the analysis of individual data revealed a correlation between rMT changes, the serological status, and the length of GFD. More in detail, even though the median decrease of rMT was almost the same for all participants, a noticeable increase in hyperexcitability was present only in those with a more extended gluten restriction and antibodies seroconversion. Antibodies were indeed still present in 7 out of 13 subjects, and most of them (5 out of 7) exhibited the smallest rMT variation [54]. This occurrence may suggest a more active role of the hyperexcitability-induced cortical plasticity in these patients, as already showed in other neurological disorders by using specific TMS mapping techniques [78,79]. In other words, the patients with long-standing gluten exposure may explain why neurological involvement persisted despite GFD. The hypothesis is supported by previous CD-associated epilepsy studies showing that the effectiveness of the diet is directly connected to the duration of neurological disorders and the age where GFD starts [80,81]. Lastly, it is important to note that a degree of improvement of depressive symptoms was observed in the study, confirming the significance of GFD in the management of CD-related neuropsychiatric symptoms [54].

### 5.4. TMS in CD after a Long Gluten-Free Diet

A recent cross-sectional TMS study after a much longer GFD (mean period of 8.5 years) found that a more extended time without consuming gluten is necessary when trying to revert the cortical changes in adult CD patients [55]. It was ascertained that an extended period of GFD was effective in restoring the cortical excitability towards a normal level. Unlike the previous study where GFD was shorter and the serum antibodies still active in a relevant part of subjects [54], in this study the two measures of global inhibition and excitation of the motor cortex (rMT and CSP, respectively) were similar to the healthy controls, suggesting a “restorative” role of a long-term diet. Nonetheless, the data also reveal that a differential behaviour of specific cortical circuits in CD may take place, with some changes responding to GFD and others that remain the same, suggesting a segregation within the synaptic plastic rearrangement phenomena [55]. Regarding serological status, although the antibodies were negative in the whole sample, the comparison of ICF in GFD patients subdivided into two groups based on their tissue tTG conversion time showed a significant gradual decrease from those with a shorter conversion time (<6 months) to those with a longer conversion time (>6 months), whose ICF was more similar to that of *de novo* patients before the diet [55]. Therefore, in patients with a shorter tTG conversion time, an enhanced ICF might play a compensatory role for a dysfunctional network within the intracortical interneurons. Another explanation is that an intracortical synaptic dysfunction, mostly involving excitatory interneurons that reflects the activity of cortico-cortical networks different from those preferentially activated by single pulse stimulation [82], may occur in CD and poorly respond to GFD [55]. 

## 6. Discussion 

### 6.1. Main Findings and Translational Value

The main translational value of this topical review is that TMS can help in monitoring and assessing CD patients, including those without a clear neurological involvement. Particularly, TMS changes are often subclinical (mostly referred as a “celiac iceberg”) and further studies require close monitoring of these patients because of the possibility of development of a clinically visible neurological syndrome in patients in different age sets (“symptomatic celiac disease”). The scope of the reported studies was indeed to demonstrate the increased risk of cognitive and neuropsychiatric complications in silent or atypical forms of CD, especially in patients with advanced age or those that are older during the diagnosis period [83]. It is important to note that despite their significant role, neuronal antigens and anti-ganglioside antibodies are not always associated with the neurological status and its complications in the course of CD [70,83,84]. 

Since the level of neurological damage associated with CD is mostly subclinical, there would be a significant burden to the brain over time. From a neurocognitive perspective, such injury may accumulate and reduce the cognitive reserve, thus predisposing to a functional decline and possibly leading to dementia, depending on the nature of the injury and how long it has been sustained. If such assumptions were correct, there would be more visible signs of cognitive or neurological impairment, or both, in older people with AGA when compared to age-matched controls. Confirmation of this hypothesis is necessary for gaining understanding about gluten as a factor that can be regulated to slow down cognitive decline not only in adults with the CD but in the general population at risk of gluten exposure. In this context, it is vital to use case-control studies to properly address the issue. 

In addition to cognitive deficits, a variety of psychiatric co-morbidities often complicates the diagnosis, reduces the quality-of-life and worsens the prognosis of celiac patients. In particular, CD can be associated with anxiety disorders, dysthymia, major depression, bipolar disorders, schizophrenia, eating disorders, autism spectrum disorders, and attention-deficit hyperactivity disorders [8,85]. TMS has been widely used to detect cortical abnormalities at a circuit level in these conditions. The results indicate a general alteration of motor cortical inhibition in different psychiatric disorders, thus providing advances in the understanding of the pathophysiology and neurochemistry underlying these conditions, although further investigations are needed to improve the therapeutic implications of TMS [38,45,86,87]. Notably, thanks to the ability to transiently modulate cortical activity, rTMS is becoming increasingly important in clinical applications for psychiatric disorders. Previous studies have already demonstrated its promising efficacy in depression and schizophrenia and emerging evidences have also been found in anxiety disorders, obsessive-compulsive disorder, and substance or food craving [88]. In particular, the efficacy in patients with medication-resistant symptoms has drawn great attention [89,90]. Nevertheless, the current literature features some conflicting results, varied quality of studies, and a lack of consensus on optimal rTMS settings [91,92].

By applying a pure neurophysiological point of view, research findings from the TMS studies here reviewed converge on a trend towards a “hyperexcitable celiac brain” and an impaired central motor conductivity [93], although it may not be restricted to the cerebral cortex [52]. With this regard, it has already been established that an overt motor cortex hyperexcitability is a relatively stable electrophysiological feature of both AD and VaD [50]. This finding has been linked to the plastic compensatory response to neuronal loss and axonal disruption respectively [94], supporting the theory that dementia is a dynamic condition and that changes of specific TMS parameters are indexes of cortical plasticity [95,96]. Notably, hyperexcitability in early AD patients was associated with deficit in long-term potentiation-like plasticity, and, therefore, there is a need to apply specific TMS paradigms (namely, the paired associative stimulation) in future studies that further investigate changes in neuroplasticity in these subjects [97]. Mechanisms of neural plasticity might offset cognitive decline and provide insight into the loss or preservation of cognitive domains. This hypothesis has been demonstrated by applying TMS mapping technique in AD patients, suggesting functional reorganization compensating for disease development since its early stages [78,98].

With regards to the humoral autoimmunity to neuronal antigens, deposits of anti-tTG2 and anti-tTG6 antibodies have been found in the small intestine and in different CNS sites (brain blood vessels, medulla, pons, cerebellum) [99]. Moreover, a possible blood-brain lesion, secondary to diffuse infiltration of T-lymphocytes and inflammatory cells within the perivascular cuffing, might leave cerebral tissues to contact with antibodies [9]. The result would be a cycle of adverse reactions that lead to a prevailing synaptic hyperexcitation and a weaker inhibition at the cortical level [53]. The increased excitability may also be related to the glutamate-induced cortical rearrangement or to a dysfunctional control involving GABAergic inhibitory interneurons. Notably, because glutamate is vital in synaptic plasticity, it can be theorized that the immune system dysregulation, triggered by gluten ingestion, resulted in a long process activation of the post-synaptic glutamate receptors accounting for the enhanced brain excitability [54,55].

### 6.2. Impact of the Gluten-Free Diet

The response of neurological symptoms to GFD is still debatable. The current knowledge includes an initial phase when patients are “gluten-sensitive” followed by a stage characterized by “gluten-insensitivity” [100]. Patients with an advanced age and prolonged use of food containing gluten explain why neurological symptoms often remain visible after a relatively short period of GFD. Furthermore, restricting the use of gluten does not yield positive results for patients with a refractory CD or in those with a co-morbid autoimmune disease or some neurological complications [8,9,101].

Here, the central question is whether a GFD will prevent or slow down the cognitive impairment in CD, which would be testable in a longitudinal study with GFD as an intervention factor. This effect is true for most, but not all, the cases of non-cognitive neurological manifestations, such as gluten ataxia, peripheral neuropathy, and epilepsy. Regarding cognition, it is within reason to state that some aspects may improve after implementing a restrictive diet while in others it may not change, supporting the theory that a more prolonged and strict GFD is likely to cause a clinical and neurophysiological remission. Nevertheless, as mentioned, considering that the neurological impairment may progress despite an adequate adherence to GFD [100,102,103,104], it is essential to consider other possible causative factors [105]: (a) the contribution of other components that are independent of GFD [52]; (b) a direct gliadin-mediated inflammatory attacks; (c) accidental minimum gluten contaminants despite a good dietary compliance [106].

### 6.3. Limitations

Even though innovative and potentially promising, the TMS-based approach to CD have to consider a number of pitfalls and critical aspects, mostly related to the technique itself: (a) as cited, TMS changes are not disease-specific and an association finding does not mean causative relationship; (b) the spatial resolution of TMS is more limited than neuroimaging, even when a specifically-designed coil is used; (c) lack of changes in TMS measures does not necessarily rule out the presence of other neuroplastic mechanisms; (d) TMS is primarily conceived for assessing M1, which is not the most involved area in patients with cognitive or neuropsychiatric disorders; therefore, generalizing findings from motor to non-motor areas requires warning, even though TMS has been shown to reliably probe the excitability and connectivity of both motor and non-motor networks [60]; (e) the few available results on relatively small sample size might not be confirmed on larger populations, even though the samples have been very homogeneous in terms of demographics, clinical, and neuroradiological features and age-matching with healthy controls.

## 7. Conclusions

To date, the development of cognitive deficit cannot be anticipated through conventional investigations. Therefore, bridging gaps within the cognitive-associated aspects of the gut-brain axis is mandatory. The best way we can currently tackle dementia is through the risk factors. In this frame, identifying new and likely modifiable risk factors, which affect a relevant proportion of the population and may feasibly be addressed through lifestyle modifications, might be of awesome importance to patients, clinicians, and researchers. Longitudinal works are warranted to establish the exact course also in non-restricted patients, as well as to assess whether there is a length-related impact associated with GFD. Similarly, studies on dementia populations that retrospectively examine serological positivity with respect to incidence and cognitive progression also are needed.

## Figures and Tables

**Table 1:** Studies using Transcranial Magnetic Stimulation (TMS) in patients with celiac disease (adapted from [93]).

Study	No. of Patients	Sex	Age (Years)	Neurological Features	Main Results	Response to the GFD	Translational Value
Pellecchia MT, et al. 1999 [51]	1	M	34	Progressive cerebellar ataxia	▼ MEPs amplitude	Partial response	Impairment of central motor pathways in CD, which partially respond to GFD
Tijssen MA, et al. 2000 [52]	2 (only 1 undergoing TMS)	M	50	Myoclonic ataxic syndrome	▲ MEPs latency in the left tibialis anterior muscle; abnormal cortical inhibition	Not reported	The enhanced excitability of sensory-motor cortex may arise as a remote effect of cerebellar pathology in CD
Pennisi G, et al. 2014 [53]	20	16 F 4 M	Median 33.0 (range 24–45)	Dysthymic disorder (5 patients); anxiety (2 patients)	▼ CSP ▼ ICI ▲ ICF	GFD was not started yet (*de novo* patients)	The immune system dysregulation might trigger changes of cortical excitability
Bella R, et al. 2015 [54]	13	10 F 3 M	Median 39.0 (range 24–46)	Dysthymic disorder (1 patient)	Compared to *de novo* patients: ▼ rMT ▼ CSP ▼ SICI ▲ ICF	Increased cortical excitability after a relatively short period of diet	Functional cortical reorganization, probably compensating for the disease progression; insufficient GFD duration
Pennisi M, et al. 2017 [55]	(a) 20 *de novo* CD patients (b) 20 CD patients on GFD	(a) 4 M 16 F (b) 6 M 14 F	(a) Mean 35.00 ± 12.03 (SD) (b) Mean 35.10 ± 6.02 (SD)	(a) Dysthymic disorder (5 patients); higher score for depression, anxiety and irritability (b) Normal	▼ CSP in *de novo* patients than GFD patients ▼ motor response amplitude in all patients ▼ SICI and enhanced ICF in all patients ▲ ICF in gluten-restricted patients compared to those non-restricted	A prolonged dietary regimen induced a recover of most but not all, the electrocortical changes	Although the clinical-neurophysiological recovery, some subtle intracortical synaptic dysfunction may persist notwithstanding the GFD

CD = celiac disease; CSP = cortical silent period; F = female; GFD = gluten-free diet; ICF = intracortical facilitation; SICI = short-latency intracortical inhibition; M = male; MEPs = motor evoked potentials; rMT = resting motor threshold; SD = standard deviation; ▼ = decrease/reduction/less prolonged; ▲ = increase/enhancement/more prolonged.

## Abbreviations

AD	Alzheimer’s disease
AGA	anti-gliadin antibodies
AMPA	α-amino-3-hydroxy-5-methyl-4-isoxazolepropionic acid
CD	celiac disease
CNS	central nervous system
CSP	cortical silent period
EEG	electroencephalography
GABA	gamma-aminobutyric acid
GAD	glutamic acid decarboxylase
GFD	gluten-free diet
HLA	human leukocyte antigen
ICF	intracortical facilitation
M1	primary motor cortex
MEP	motor evoked potential
NMDA	*N*-methyl-d-aspartate
rMT	resting motor threshold
rTMS	repetitive transcranial magnetic stimulation
S1	primary somatosensory cortex
SICI	short-latency intracortical inhibition
TMS	transcranial magnetic stimulation
tTG	tissue transglutaminase
VaD	vascular dementia
